# International Szent-Györgyi Prize for Progress in Cancer Research: basic and translational research recognition: Mary-Claire King received the 2016 Prize for her pioneering research that demonstrated the first evidence of genetic predisposition to breast cancer

**DOI:** 10.1186/s40880-017-0258-8

**Published:** 2017-11-21

**Authors:** Hali Hartmann, Jie Zhao, Sujuan Ba

**Affiliations:** National Foundation for Cancer Research, 4600 East West Highway, Suite 525, Bethesda, MD 20814 USA

**Keywords:** The National Foundation for Cancer Research, The Szent-Györgyi Prize, Mary-Claire King, *BRCA1* and genetic testing

## Abstract

The Szent-Györgyi Prize for Progress in Cancer Research is a prestigious scientific award sponsored by the National Foundation for Cancer Research (NFCR)—a leading cancer research charitable organization in the United States that supports innovative cancer research globally with the ultimate goal to cure cancer. The coveted Szent-Györgyi Prize annually honors a scientist whose seminal discovery or body of work has resulted in, or led toward, notable contributions to cancer prevention, diagnosis, or treatment; and the discovery has had a high direct impact of saving people's lives. In addition, the prize promotes public awareness of the importance of basic cancer research and encourages the sustained investment needed to accelerate the translation of these research discoveries into new cancer treatments. In 2016, NFCR’s Szent-Györgyi Prize Selection Committee was unanimous in its decision to recognize an icon in human disease genetics, Dr. Mary-Claire King, for her pioneering research that demonstrated the first evidence of genetic predisposition to breast cancer. Her proof of existence of *BRCA1* gene and its location has made genetic screening for breast and ovarian cancers possible, saving lives of many people who are at high risk with inherited *BRCA1* mutations.

The Szent-Györgyi Prize for Progress in Cancer Research, named in honor of Albert Szent-Györgyi, M.D., Ph.D., 1973 Nobel laureate and co-founder of the US National Foundation for Cancer Research (NFCR), is awarded annually to outstanding research scientists whose basic and translational research achievements have expanded our understanding of cancer and cancer causation; whose vision has moved cancer research in new directions; and whose discoveries have resulted in notable advances in cancer prevention, diagnosis, or treatment.

We have previously reported in the *Chinese Journal of Cancer* on the two preceding Szent-Györgyi Prize recipients, the 2014 Prize winner James P. Allison, Ph.D. [1] and the 2015 Prize winner Frederick W. Alt, Ph.D. [2], and this Prize has now been given to 12 recipients since its inauguration in 2006 for these recipients’ discoveries that have changed the course of cancer research with an impact of saving people’s lives. The international scope of the Prize was first exemplified when the 2012 Selection Committee awarded the Prize to co-recipients from China, Zhu Chen, M.D., Ph.D., the Chairman of the Chinese Medical Association and Former Minister of Health of China, and his mentor, Zhen-Yi Wang, M.D.

The 2016 Szent-Györgyi Prize was awarded via unanimous decision by the 2016 Selection Committee to Mary-Claire King, Ph.D., Professor of Medicine (Medical Genetics) and Genome Sciences at the University of Washington in Seattle, for her pioneering research that provided the first evidence of genetic predisposition to breast cancer (Fig. 1) [3]. Her proof of existence of the breast cancer 1 (*BRCA1*) gene and the identification of its location made genetic screening for breast and ovarian cancers possible, giving individuals who have inherited mutations in *BRCA1* a chance to take preventive measures at an early stage of their lives.

**Fig. 1:**
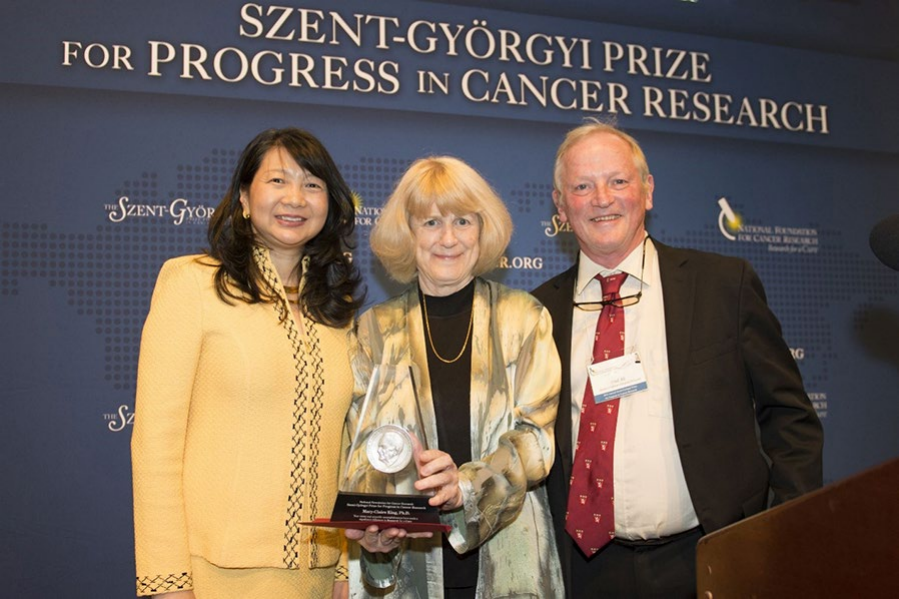
The 2016 Szent-Györgyi Prize Ceremony. Celebrating Dr. Mary-Claire King’s receipt of the Prize, from left to right: Dr. Sujuan Ba, Co-chair of the 2016 Szent-Györgyi Prize Selection Committee and President of the National Foundation for Cancer Research (NFCR); Dr. Mary-Claire King, winner of the 2016 Szent-Györgyi Prize; and Dr. Frederick W. Alt, winner of the 2015 Szent-Györgyi Prize and Chair of the 2016 Prize Selection Committee

## Mary-Claire King: an extraordinary geneticist

In her Ph.D. thesis (under the late Allan Wilson of University of California, Berkley), Dr. King began her remarkable career demonstrating that human and chimpanzee are 99% identical in coding sequences of genes (validated 30 years later when the chimpanzee genome was published). Proposing that the differences are due to a small number of genetic variations that affect gene regulation and timing of gene expression during development, King and Wilson’s regulatory hypothesis remains one of the central paradigms of human evolution [4]. In 1971, when the US President Richard Nixon declared the “war on cancer,” a big unanswered question about breast cancer was that it was increasing (as Dr. King noted, we know now this increase was not for reasons of genetics), and it was clearly in families. Mary-Claire King asked the question if the familial clustering of breast cancer could really be due to inherited genetics. Her thoughts were further influenced by earlier literature including the work of Dr. Paul Broca, a mid-nineteenth century French physician who was first to accurately and systematically describe familial breast cancer. As an example, his wife had breast cancer and noting the cancer spanned four generations in her family—he articulated that the cancer can be inherited [5]. By the 1970s, it had been hypothesized that the malignancies such as asbestos-related lung cancer, bladder cancer, and colon cancer were due to environmental causes; but breast cancer was viewed as a common, complex disease that may arise from interactions among multiple genes and environmental causes. The idea that cancer was fundamentally a genetic disease was not widely accepted. As an evolutionary biologist, Dr. King reasoned that she could take the same approaches used in her dissertation thesis to try to test what might be the case on an extremely short evolutionary time scale, a time scale not of 5 million years (as the case with man and chimpanzee), but of a couple of generations. To answer the question of whether breast cancer could be genetically inherited, she needed a population, a database for which to model and apply statistical analysis. Dr. King had a fortunate opportunity to work with the US National Cancer Institute (NCI) on their ongoing highly significant study on menopausal estrogens and oral contraceptives and risk of breast, ovarian, and endometrial cancers. During her interview with the participants, she was able to ask these women additional questions about their family history and eventually records of more than 1500 breast cancer patients were collected.

In 1988, Dr. King and her team published their data which, when applied to a maximum likelihood statistics model, concluded that by estimation, about 4% of breast cancer cases were directly linked to inherited mutations in a hypothetical gene. Her genetic analysis featured that the inheritance was autosomal dominance and that for tumor initiation to begin, there would be a second hypothetical hit in the same gene in a breast cell. Among women carrying mutations in the gene, the risk of breast cancer by age 70 was 82%; in contrast, among women without a susceptibility allele, the risk of breast cancer by age 70 was 8% [6].

Proving her model required families in which such a gene could be identified. The *BRCA1* gene project was founded when Dr. King began working with 23 severely affected families, characterized by multiple cases of early onset invasive breast cancer—many of whom she has now known for over 40 years.

Mary-Claire King was the first to exploit DNA linkage studies to uncover a gene for a complex disease. During the 17 years from 1974 to 1990, she and her small team worked with human geneticists from around the world—each of whom had their own individual phenotypes of interest—to identify markers in all chromosomes that could be used to trace chromosomal segments. They all agreed to genotype every marker that was developed.

In 1990, Dr. King and her team published their work that a gene on a section of chromosome 17—which they named *BRCA1*—carried specific markers in women with breast cancer in the most severely afflicted families [7]. As she remarked in her address at the Szent-Györgyi Prize ceremony, “the goodness of fit of co-segregation of our little set of markers on chromosome 17 with breast cancer and a few ovarian cancers in each of these families with the youngest ages of onset (was) incredibly well, and you know they were not perfect, as it turned out that recombination matters. But until we get to an average age of onset of about age 45, the goodness of fit was fantastic, I mean we had odds 10^6^–1 in favor of co-segregation, and you don’t get 10^6^–1 with seven families by chance.”

Dr. King subsequently showed that the risks of breast and ovarian cancers among women with *BRCA1* mutations are very high, up to a lifetime risk of 80% for breast cancer and greater than 40% lifetime risk for ovarian cancer [8]. The research community has elucidated that *BRCA1* is a tumor suppressor gene with one of its functions to repair damaged double-stranded DNA, and more than 1600 mutations have been detected in it to render most of which lead to a missing or faulty protein [9]. Subsequently, another major breast cancer- and ovarian cancer-associated gene, *BRCA2*, was identified by another group in 1996 [10].

Dr. Mary-Claire King’s scientific rigor, persistence, and dedication have made genetic screening methods available to identify people at high risk. Subsequently, preventative and therapeutic approaches have been developed to treat breast and ovarian cancers more effectively.

Dr. King and her colleagues recently developed a widely used clinical screening platform (which she named BROCA in honor of Dr. Paul Broca) that includes multi-gene capture and parallel sequencing tools to detect all the mutated genes that have been discovered that predispose women to breast and/or ovarian cancer [11].

“And what I am now advocating for is that all women, regardless of personal or family history, be offered complete sequencing of *BRCA1* and *BRCA2* when they are 30 years old, that it would be comparable to a pap smear but you only have to do it once,” stated Dr. King as she closed her address to the audience.

“What we care about are slam dunk mutations that absolutely are associated with enormously reduced risks of mortality if a woman undertakes prophylactic salpingo-oophorectomy by the age of 40 and for many women, prophylactic mastectomy, usually sometime thereafter.”

“…There is no reason now that any woman with a mutation in *BRCA1* or *BRCA2* should ever die of breast cancer or ovarian cancer. And it is my goal to see that that’s the case.”

**Fig. 2:**
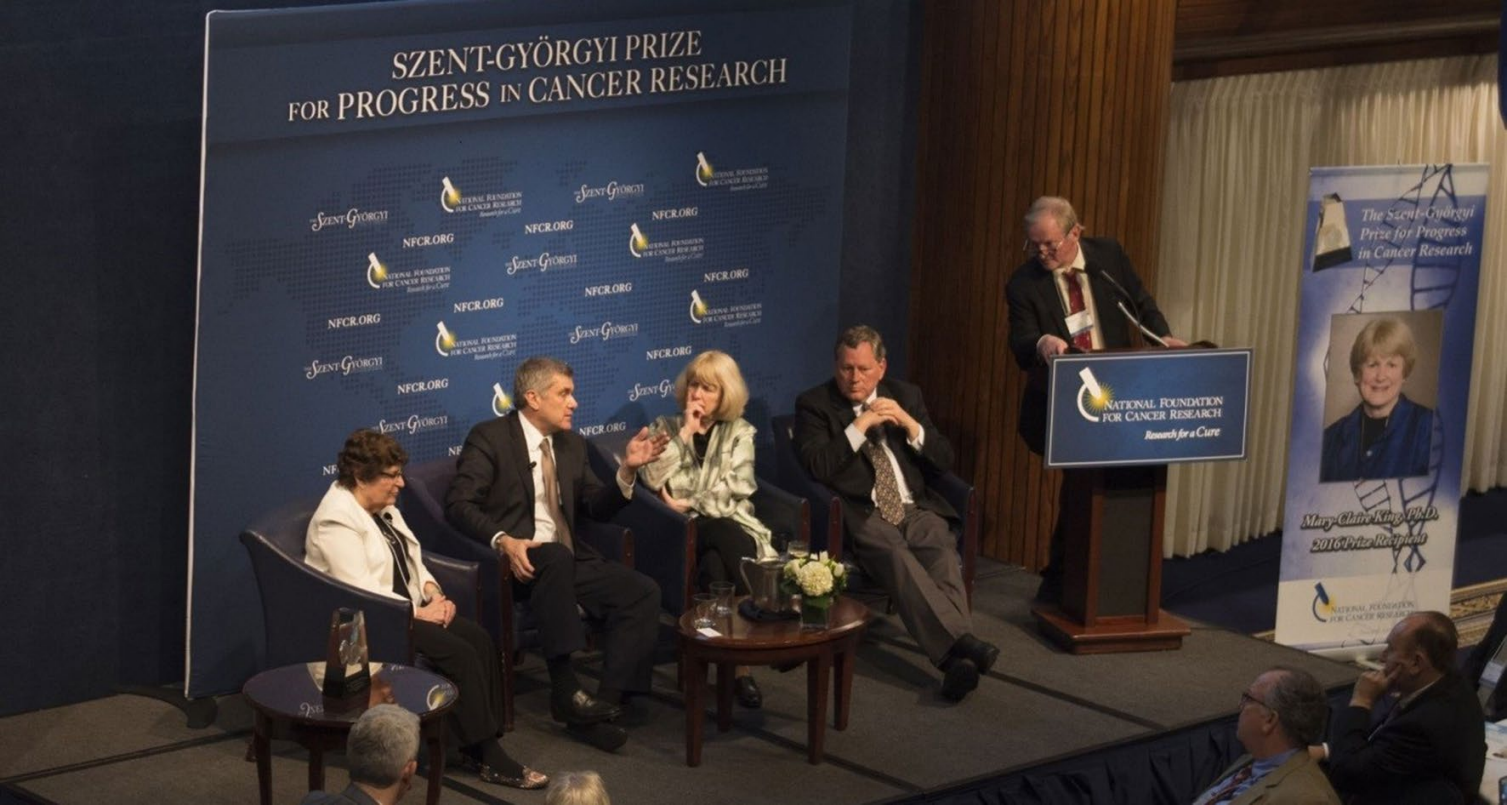
The inaugural Szent-Györgyi Prize Panel Discussion. Panelists from left to right: Susan Band Horwitz, Ph.D.; Craig B. Thompson, M.D.; Mary-Claire King, Ph.D.; Webster Cavenee, Ph.D., and at the podium, Frederick W. Alt, Ph.D

The Inaugural Szent-Györgyi Prize Panel Discussion with audience participation was held following Dr. King’s address to encourage more in-depth discussions on the widespread genetic testing applications. Joining Dr. King on the panel were renowned cancer research leaders, Webster Cavenee, Ph.D., Director of Strategic Alliances CNS at Ludwig Institute for Cancer Research and Distinguished Professor of University of California, San Diego; Susan Band Horwitz, Ph.D., Distinguished Professor and Rose C. Falkenstein Chair in Cancer Research at Albert Einstein College of Medicine; and Craig B. Thompson, M.D., President and CEO of the Memorial Sloan Kettering Cancer Center. Panel moderator was Frederick W. Alt, Ph.D., Charles A. Janeway Professor of Pediatrics and Professor of Genetics at Harvard Medical School, Director, Program in Cellular and Molecular Medicine at Boston Children’s Hospital (Fig. 2). The prestigious Szent-Györgyi Prize continues to showcase how basic and translational cancer research will generate 21st century precision medicine—the future hope and promise for effective, individualized patient options.
